# Oscillatory electroencephalographic patterns of arithmetic problem solving in fourth graders

**DOI:** 10.1038/s41598-021-02789-9

**Published:** 2021-12-02

**Authors:** Clemens Brunner, Nikolaus A. Koren, Judith Scheucher, Jochen A. Mosbacher, Bert De Smedt, Roland H. Grabner, Stephan E. Vogel

**Affiliations:** 1 Institute of Psychology, Educational Neuroscience, University of Graz, Graz, Austria; 2 Parenting and Special Education Research Unit, KU Leuven, Leuven, Belgium

**Keywords:** Neuroscience, Cognitive neuroscience, Problem solving

## Abstract

Numerous studies have identified neurophysiological correlates of performing arithmetic in adults. For example, oscillatory electroencephalographic (EEG) patterns associated with retrieval and procedural strategies are well established. Whereas fact retrieval has been linked to enhanced left-hemispheric theta ERS (event-related synchronization), procedural strategies are accompanied by increased bilateral alpha ERD (event-related desynchronization). It is currently not clear if these findings generalize to children. Our study is the first to investigate oscillatory EEG activity related to strategy use and arithmetic operations in children. We assessed ERD/ERS correlates of 31 children in fourth grade (aged between nine and ten years) during arithmetic problem solving. We presented multiplication and subtraction problems, which children solved with fact retrieval or a procedure. We analyzed these four problem categories (retrieved multiplications, retrieved subtractions, procedural multiplications, and procedural subtractions) in our study. In summary, we found similar strategy-related patterns to those reported in previous studies with adults. That is, retrieval problems elicited stronger left-hemispheric theta ERS and weaker alpha ERD as compared to procedural problems. Interestingly, we observed neurophysiological differences between multiplications and subtractions within retrieval problems. Although there were no response time or accuracy differences, retrieved multiplications were accompanied by larger theta ERS than retrieved subtractions. This finding could indicate that retrieval of multiplication and subtraction facts are distinct processes, and/or that multiplications are more frequently retrieved than subtractions in this age group.

## Introduction

Mathematical ability^1^ is a key competence in modern societies, which is positively correlated with adult socioeconomic status^2^. Building on basic numerical skills, arithmetic (the study of numbers and their operations such as addition, subtraction, multiplication, and division) is one of the first mathematical concepts taught in primary schools. Therefore, understanding the mental substrates and neurocognitive mechanisms of arithmetic has been an important line of interdisciplinary research over the past decades^3–6^. This study further investigates these mechanisms in children using electrophysiological oscillatory activity.

Adults generally use either procedures or retrieval from memory to solve arithmetic problems^7^. Whereas procedures comprise a variety of different strategies that evolve with practice and age (such as counting all operands, counting from the larger of two operands or decomposing a problem into a series of simpler problems), solving a problem via retrieval directly accesses the solution from long-term memory. Over development, sufficient practice eventually leads to a shift from procedural strategies to more efficient fact retrieval^8–12^ and automatic rule-based procedures, such as those for multiplications involving zero (0 × *N* = 0) or one (1 × *N* = *N*)^13^.

Typically, adults solve easy problems more often using retrieval and hard problems more often using procedures^7, 14, 15^. Easy and hard problems are often characterized by their problem size, which is related to the magnitude of the involved numbers. More precisely, small problems with small operands and solutions are considered easy problems (e.g., 6 − 2 = 4), whereas large problems with large operands and solutions are deemed hard problems (e.g., 32 − 6 = 26). In general, people solve small problems fast and accurately, while they take longer and make more errors with large problems. Notably, response times as well as error rates continuously increase with problem size. This so-called problem size effect is one of the most robust findings in the field of mathematical cognition^16^.

Because adults solve small problems primarily via fact retrieval and large problems primarily via procedures, many studies have used problem size as a proxy for strategy use^12, 17^. However, this approach disregards variability in strategy use within and between individuals^18^. Importantly, strategy use also changes with age^12^, so a given problem size categorization tailored for adults cannot be directly transferred to children. Individual strategy reports can mitigate these issues. Although such subjective reports can be biased if instructions are not carefully designed^19^, they can give a more accurate representation of individual strategy use when used correctly, especially when assessed on a trial-by-trial basis^20, 21^.

To date, the majority of neurophysiological studies on performing arithmetic have used functional magnetic resonance imaging (fMRI)^22, 23^. These studies provide converging evidence that performing arithmetic recruits a widespread network involving prefrontal, posterior-parietal, occipito-temporal, and hippocampal areas^24^. More specifically, several fMRI studies reported distinct neural activation patterns for retrieval and procedural strategy use^25–27^. Whereas retrieving arithmetic facts from memory is associated with stronger activation of the left angular gyrus, procedural strategies are associated with increased activation in a widespread fronto-parietal network^25^. Although these networks are superficially similar in children and adults, there are notable differences. For example, children show increased hippocampal activity related to retrieving arithmetic facts from memory^23, 28^, which is typically not present in adults^29^.

In comparison, relatively few studies have used electroencephalography (EEG) or magnetoencephalography (MEG) to study arithmetic processes in adults^30^ and children^23^. However, EEG/MEG and fMRI are often viewed as complementary techniques, because EEG/MEG offer excellent temporal but limited spatial resolution, whereas fMRI has excellent spatial but limited temporal resolution^31^. Moreover, EEG equipment is relatively inexpensive, portable, and can be used more easily with children even outside of laboratories^5^. For these reasons, we decided to use EEG to assess neurophysiological patterns of arithmetic in children.

Stimulus-related EEG patterns can be classified into evoked (phase-locked) and induced (non-phase-locked) activity^32^. Whereas event-related potentials capture evoked activity^33^, event-related desynchronization and synchronization (ERD/ERS) describe induced oscillations in various predefined frequency bands^34^. In general, ERD/ERS reflect dynamic changes in thalamo-cortical information processing networks^35^. Previous studies associated theta ERS (around 3–6 Hz) with information retrieval from memory^36, 37^ and working memory^38^. In contrast, alpha ERD (around 8–12 Hz) is associated with increased task difficulty, more efficient task performance, and increased effort and attention^35, 39^.

A number of EEG studies in both adults and children have reported correlates of arithmetic strategy use in evoked^40–43^ and induced^17, 21, 44, 45^ EEG activity. In particular, several studies in adults observed left-hemispheric theta ERS with small (or self-reported retrieved) problems and bilateral posterior alpha ERD with large (or self-reported procedural) problems^17, 21, 46–48^. These findings provide evidence that solving easy problems (predominantly by fact retrieval) and hard problems (mainly with procedures) involve distinct neural processing mechanisms.

Only few studies have investigated strategy-related ERD/ERS patterns in children. Among these, a study with children in fifth grade used ERD/ERS to assess strategy-related changes after multiplication training^44^. The authors found decreased alpha ERD for trained versus untrained problems, which they interpreted as a shift towards more efficient automated procedural or retrieval processes. A follow-up study found increased theta ERS for trained problems (again with fifth graders), which the authors regarded as further evidence for an increased use of automated procedures and retrieval strategies after training^45^.

In addition to investigating strategies, several studies have also identified differences between arithmetic operations^17, 26–28, 49–57^. Clearly, operation effects are influenced by strategy use, problem size, and individual learning history^23, 58^. In general, adults solve multiplications predominantly by retrieval, whereas subtractions typically also involve procedures^58^. Therefore, it is important to take individual strategy use into account when analyzing cognitive mechanisms associated with arithmetic operations^26^. For example, Polspoel et al. found strategy-related activation patterns similar to those present in adults, but no operation differences after accounting for strategy use in their fMRI study with fourth graders^27^. However, some studies provide evidence that retrieving arithmetic facts involves distinct operation-specific memory networks^59–61^. This interaction of strategy and operation effects might therefore be observed as neurophysiological differences between operations only within fact retrieval problems (for example between multiplication and subtraction facts).

The present study is the first to investigate ERD/ERS correlates of both strategy use and arithmetic operations in children. Based on the finding that retrieval and procedural strategies are associated with distinct ERD/ERS patterns in adults, one goal in this study was to investigate if similar patterns exist in children. We also wanted to provide more insights into a potential interaction of strategy and operation on ERD/ERS and identify any effects that cannot be found on a behavioral level. We decided to focus on children in a narrow age range of 9–10 years (fourth graders), because they have already studied and practiced arithmetic for several years. In particular, children in Austrian elementary schools typically start to learn multiplication tables (up to 10 × 10) and practice subtractions (involving numbers up to 100) in second grade.

Against this background, our study aims to assess ERD/ERS patterns related to strategy use (retrieval versus procedural) and operations (multiplication versus subtraction) in fourth graders. We expected similar strategy-related behavioral patterns as found in adults, namely faster response times and higher solution accuracies for retrieval as compared to procedural strategies. Likewise, we also expected similar strategy-related ERD/ERS patterns—that is, increased theta ERS for retrieval problems and increased alpha ERD for procedural problems. Given the inconclusive findings regarding operation differences, we did not have a clear hypothesis whether we would find neurophysiological differences between multiplications and subtractions. On the one hand, the fMRI study by Polspoel et al. with a similar design did not identify distinct activation patterns between these operations after accounting for strategy use^27^. On the other hand, due to its excellent temporal resolution, ERD/ERS might be more sensitive to subtle differences between operations that are clearly reflected in response time differences in that study, specifically within fact retrieval problems.

## Methods

### Participants

A total of 36 children (18 female and 18 male, aged between nine and ten years) participated in our study. They all attended fourth grade of elementary schools in or near Graz, Austria. All of them were native German speakers. However, we discarded five participants for the following reasons:

- one child had a neurological disease,
- two children solved none or only one out of 20 large multiplication problems correctly,
- all EEG channels in a region of interest were noisy in one child,
- and audio data from one child was corrupted so we could not evaluate response times.

Therefore, the final sample consisted of 31 children (13 female and 18 male, mean age 10.1 years with a standard deviation of 0.5 years). Prior to their participation, both children and their parents or legal guardians gave informed consent to take part in the study. Each child received a 30€ gift card from a local toy store. The study was approved by the ethics committee of the University of Graz and all experiments were performed in accordance with relevant guidelines and regulations.

### Stimuli

Each child solved 80 unique arithmetic problems (40 subtractions and 40 multiplications). We presented these problems in a sequence of four blocks (A, B, C, D) separated by short breaks. The order of the blocks was counter-balanced across participants (A, B, C, D and C, D, A, B), but the pseudo-randomized order of problems within each block was the same for all participants.

Table 1 lists the whole problem set (adapted from Polspoel et al.^27^). Each block contains five small subtractions (e.g., 6 − 2), five small multiplications (e.g., 9 × 2), five large subtractions (e.g., 36 − 8), and five large multiplications (e.g., 16 × 6). Both operands are less than or equal to ten in small problems, whereas one operand is greater than ten in large problems. In addition, the subtrahend in large subtractions is always greater than the ones digit of the minuend. Polspoel et al.^27^ designed and validated this problem set so that small problems are likely to be solved by retrieval and large problems are likely to require a procedure.

**Table 1:** Problem set consisting of 40 subtractions and 40 multiplications presented in four blocks.

A	B	C	D
6 − 2	36 − 8	3 × 14	16 × 6
4 − 3	3 × 16	8 − 2	33 − 9
9 × 2	7 − 3	23 − 8	8 × 2
26 − 7	9 − 7	4 × 5	8 − 5
5 × 4	22 − 6	4 − 2	3 × 12
35 − 8	5 × 3	4 × 12	14 × 5
5 × 13	38 − 9	7 × 3	7 − 2
9 − 2	3 × 3	28 − 9	12 × 5
12 × 6	4 × 15	6 − 4	9 − 4
7 − 5	31 − 8	2 × 4	4 × 13
24 − 9	6 × 2	27 − 8	5 − 3
2 × 2	25 − 8	15 × 3	24 − 5
5 × 15	3 − 2	33 − 6	10 − 4
32 − 6	15 × 6	6 × 3	3 × 2
3 × 4	4 × 4	10 − 9	34 − 6
21 − 8	3 × 7	13 × 3	2 × 7
16 × 4	6 × 14	8 − 4	37 − 8
6 − 3	5 − 2	31 − 9	8 × 3
14 × 4	13 × 6	5 × 16	26 − 8
6 × 4	10 − 2	2 × 5	5 × 5

Before the actual experiment started, children solved 12 practice trials (three problems in each of the four categories) to get familiar with the procedure. These problems were not part of the final set.

### Paradigm

**Figure 1:**
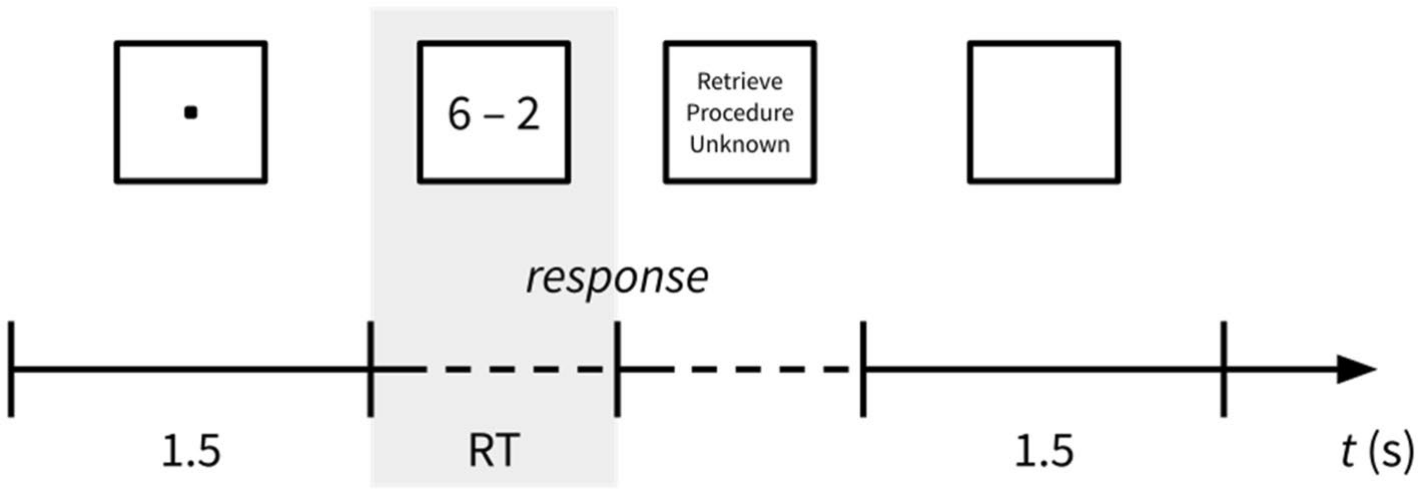
Timing of a trial. After showing a fixation dot for 1.5 s, an arithmetic problem appeared and stayed on the screen until participants verbalized their answer. We defined the time from problem presentation until answering as the response time (RT) (shaded in gray). Following the response, three strategies (retrieve, procedure, unknown) appeared, and participants verbally reported the strategy they had used. Finally, a blank screen lasting for 1.5 s concluded the trial.

Figure 1 illustrates the timing of a trial. First, a fixation dot appeared for 1.5 s, which was followed by the arithmetic problem. We instructed children to answer as quickly and accurately as possible. The problem remained on the screen until participants verbalized their solution; this defines the response time (RT). After that, a selection of three strategies (retrieve, procedure, unknown) appeared on the screen. Participants reported the strategy they used to solve the problem verbally on a trial-by-trial basis. Neither the problem calculation nor the strategy report phase had a time limit. Finally, a blank screen appearing for 1.5 s marked the end of a trial. We used the Python-based open source PsychoPy package^62^ for stimulus presentation.

### Measurement setup

Participants sat in a comfortable chair in front of a 24-inch full HD (1920 × 1080) flat screen with a refresh rate of 120 Hz. A studio-grade microphone recorded verbal responses for each trial, and we manually logged if the given answer was correct or incorrect.

**Figure 2:**
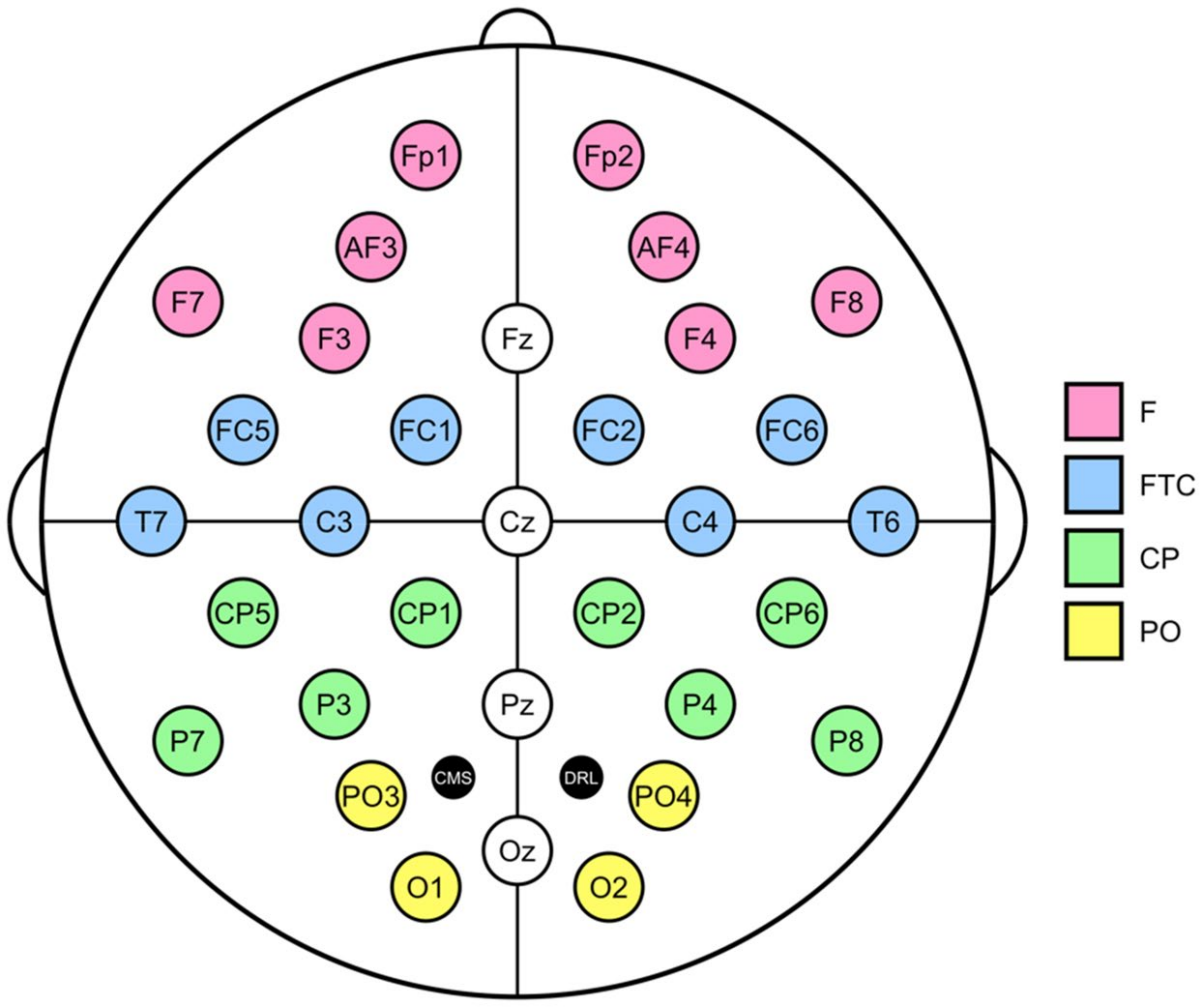
Schematic configuration of 32 EEG channels arranged in an extended 10–20 layout. Colors indicate four regions of interest (ROIs) per hemisphere: frontal (F, pink), fronto-temporo-central (FTC, blue), centro-parietal (CP, green), and parieto-occipital (PO, yellow). Midline channels Fz, Cz, Pz, and Oz were not part of any region of interest. CMS and DRL indicate locations of reference and ground electrodes, respectively.

In addition to behavioral measures, we recorded the ongoing electroencephalogram (EEG) with a BioSemi ActiveTwo amplifier using 32 channels arranged in an extended 10–20 layout over the whole scalp (see Fig. 2). The amplifier recorded data with a sampling frequency of 512 Hz and a low-pass filter of 104 Hz. Locations of reference (CMS) and ground (DRL) electrodes are indicated in Fig. 2.

### Procedure

We collected data in individual test sessions in our lab. At least two experimenters were present during each session, and a parent or legal guardian accompanied the child during the procedure. We started each session with a detailed introduction of the study, where we presented a *photo book* that explained the study, the task, and the basics of EEG in a child-friendly way. We explained the reasoning behind each step and encouraged children to ask questions. After completing the photo book, we conducted a scripted interview to ensure that all children received identical instructions. In particular, we made sure that children understood the difference between fact retrieval and procedural problem-solving strategies by giving examples for both strategies. They could also respond with “I don’t know” if they were not sure which strategy they had used to solve a given problem. To minimize possible social desirability bias, we ensured that children understood that none of the strategies were preferred over the other. Next, we discussed each step that children had to perform in the study, where we mentioned the importance of verbalizing the solution without using any filler words. We instructed children to solve each problem both as accurately and quickly as possible.

After obtaining written informed consent by children and their legal guardians, we donned the head cap and EEG electrodes. The children observed their ongoing EEG and we let them experiment with different kinds of movement to interactively experience the effects on the recorded signals. That way we made sure they understood the importance of restricting any movement during the problem-solving phase. Next, children completed 12 practice trials to familiarize themselves with the task. We then provided feedback on verbal responses (in particular regarding the volume and use of filler words) and compliance with restricting movement during the problem-solving stage.

Parents left the lab and waited in an adjacent room. Finally, we started the main experiment. The duration of the whole session was about 1 h (including task instructions as well as setting up and removing EEG electrodes).

### Data analysis

#### Strategy reports

In all subsequent analyses, we included only trials with consistent problem size and individual verbal strategy reports (given that problem size is a proxy for strategy). That is, we retained only small retrieval and large procedural problems and discarded all other trials (including trials reported to be solved with an unknown strategy). To quantify the degree of agreement between problem size and strategy report categorizations, we used Cohen’s κ coefficient^63^.

#### Behavioral data

First, we analyzed the solution accuracy (ACC) of each participant, which is defined as the number of correct responses in relation to the total number of problems per category.

Second, we defined response time (RT) as the time it took children to verbalize a solution to a given arithmetic problem. For each individual verbal response (recorded as a sound file), we determined the exact onset using the aubio package with the default high frequency content onset detection algorithm. In addition, we verified the correctness of onset detections by visual and auditory inspection. All subsequent RT and ERD/ERS analyses are based only on correct answers.

#### EEG processing

We used *MNE-Python*^64^ in custom Python scripts to process EEG data. The first step in our pipeline was to identify bad channels (for example, channels that were extremely noisy or almost completely flat, or channels containing large artifacts over the majority of their time course). We visually inspected power spectral densities and time courses of EEG signals to remove such bad channels. At least two authors independently carried out this channel selection process, and we resolved ambiguities by discussing problematic channels separately.

After dropping bad channels, we re-referenced the remaining data channels to their common average. Supported by visual indications of trial start and end points, we manually marked segments containing artifacts (such as muscle activity or electrode movement) in the continuous data. All subsequent analysis steps automatically ignore these segments.

To remove (or at least minimize) ocular artifacts (such as eye movements and blinks), we performed independent component analysis (ICA) on the remaining clean data channels and segments^65^. Because ICA is susceptible to low-frequency drifts^66^, we first applied a high-pass filter with a passband edge of 1 Hz. Using topographic plots, time courses, and power spectral densities of the resulting independent components, we manually identified those components that represented ocular activity^67^. We found between one and three ocular components for each data set. Finally, we zeroed out all excluded components and projected the original data back from component space to electrode space.

#### ERD/ERS calculation

Using clean EEG data, we computed band power in three pre-defined frequency bands by applying a suitable band-pass filter to the continuous signals and squaring each sample. Specifically, we used theta (3–6 Hz), lower alpha (8–10 Hz), and upper alpha (10–13 Hz) frequency bands^21^. Within each frequency band, we computed the median band power values of baseline and activity time intervals for each trial and channel (horizontal averaging). The baseline interval coincides with one second in the fixation period (− 1.25 s to − 0.25 s relative to problem onset), whereas the activity interval corresponds to the time segment during which participants worked on the arithmetic problem (shaded area in Fig. 1, starting with problem onset and lasting for a variable duration corresponding to individual response times). This procedure resulted in two band power values (baseline and activity) per frequency band (theta, lower alpha, and upper alpha), trial (maximum of 80), and channel (maximum of 32) for each participant (31 in total).

Based on average baseline (*B*) and activity (*A*) band power values from individual trials, we computed ERD/ERS values according to the following equation^34^:

$$\text{ERD/ERS}= \frac{{\text{A}}-{\text{B}}}{\text{B}}\times {100\%}\text{.}$$

Negative values reflect decreased band power relative to baseline (ERD), whereas positive values indicate increased band power relative to baseline (ERS). We computed the median baseline and activity band power values for trials grouped by operation and strategy (vertical averaging). Finally, based on previous studies^17, 21, 46, 68^, we aggregated ERD/ERS values from neighboring channels within four regions of interest (ROIs) and two hemispheres using the arithmetic mean. Figure 2 illustrates these regions with different colors, which roughly correspond to frontal, fronto-temporo-central, centro-parietal, and parieto-occipital regions in both hemispheres. All in all, this resulted in a total of 2,976 ERD/ERS values (corresponding to all combinations of 31 participants, two operations, two strategies, three frequency bands, four ROIs, and two hemispheres).

#### Statistical analyses

We computed descriptive statistics on strategy use, problem size, RT, ACC, and ERD/ERS as well as inferential statistics for these outcomes using appropriate (generalized) linear mixed-effects models in R. Specifically, we used a generalized linear mixed-effects model with an inverse Gaussian error distribution and identity link function to analyze RTs, because these typically have a right-skewed distribution^69^. We included two fixed effects (operation and strategy) and one random effect (participant). Similarly, we modeled ACC data with a generalized linear mixed-effects model with a binomial error distribution and a logit (log-odds) link function. This model incorporated the same fixed and random effects as the RT model. Finally, we used standard linear mixed-effects models to analyze ERD/ERS data in each of the three frequency bands (theta, lower alpha, and upper alpha) separately. We included four fixed effects (operation, strategy, ROI, and hemisphere) and one random effect (participant).

We used the R package afex, which is based on lme4^70^ and lmerTest^71^, to compute (generalized) linear mixed-effects models including *p*-values.

## Results

### Strategy reports

Table 2 illustrates the agreement between problem size and strategy report categorizations.

**Table 2:** Number of trials within problem size (rows) and strategy report (columns) categories for all participants.

	Procedure	Retrieval	Unknown
Large	1080	144	16
Small	148	1076	16

We dropped 16 + 16 = 32 problems that children reported to have solved with an unknown strategy. The remaining problem size and strategy reports are relatively consistent with an agreement proportion (accuracy) of 0.88 and Cohen’s κ of 0.76. Nevertheless, we only included the 1080 + 1076 = 2156 problems that agreed in their strategy report and problem size (i.e., small retrieval and large procedural problems) in subsequent analyses. We refer to these two problem groups as retrieval and procedural strategies, respectively. In addition, we used only correctly solved problems in our RT and ERD/ERS analyses, which further reduces the total number of problems to 2046. Table 3 summarizes the distribution of correctly solved and consistent problems per participant.

**Table 3:** Summary statistics for the number of correctly solved and consistent problems per participant (q_1_ and q_3_ denote the first and third quartiles, respectively).

Strategy	Operation	Min	*q*_1_	Median	Mean	*q*_3_	Max
Retrieve	−	6	17.0	19	17.58	20	20
Retrieve	×	7	14.5	17	16.55	19	20
Procedure	−	6	12.0	16	14.90	18	20
Procedure	×	12	14.5	18	16.97	19	20

### Behavioral data

#### Solution accuracy

Table 4 lists arithmetic means and standard deviations of solution accuracies grouped by strategy and operation. In this table and throughout the remaining manuscript, we use − and × to refer to subtraction and multiplication problems, respectively.

**Table 4:** Mean and standard deviation of solution accuracies for the four problem groups.

Strategy	Operation	Mean	Standard deviation
Retrieve	−	0.980	0.054
Retrieve	×	0.978	0.047
Procedure	−	0.910	0.100
Procedure	×	0.915	0.092

The generalized linear mixed-effects model yielded a significant main effect of strategy (*χ*^2^(1) = 56.12, *p* < 0.001), which confirms our hypothesis that children solved retrieval problems more accurately (0.982) than procedural problems (0.913). There was no significant difference between multiplication and subtraction problems (*χ*^2^(1) = 0.39, *p* = 0.534) and no significant interaction (*χ*^2^(1) = 0.32, *p* = 0.571).

#### Response time

Table 5 provides summary statistics (minimum, first quartile, median, mean, third quartile, and maximum) for correct RTs grouped by strategy and operation.

**Table 5:** Summary statistics for RT (s) (q_1_ and q_3_ denote the first and third quartiles, respectively).

Strategy	Operation	Min	*q*_1_	Median	Mean	*q*_3_	Max
Retrieve	−	0.88	1.44	1.78	2.07	2.24	10.38
Retrieve	×	0.92	1.46	1.83	2.18	2.49	15.06
Procedure	−	1.27	3.51	5.17	6.44	7.56	36.52
Procedure	×	2.28	5.59	7.98	10.05	11.48	95.23

**Figure 3:**
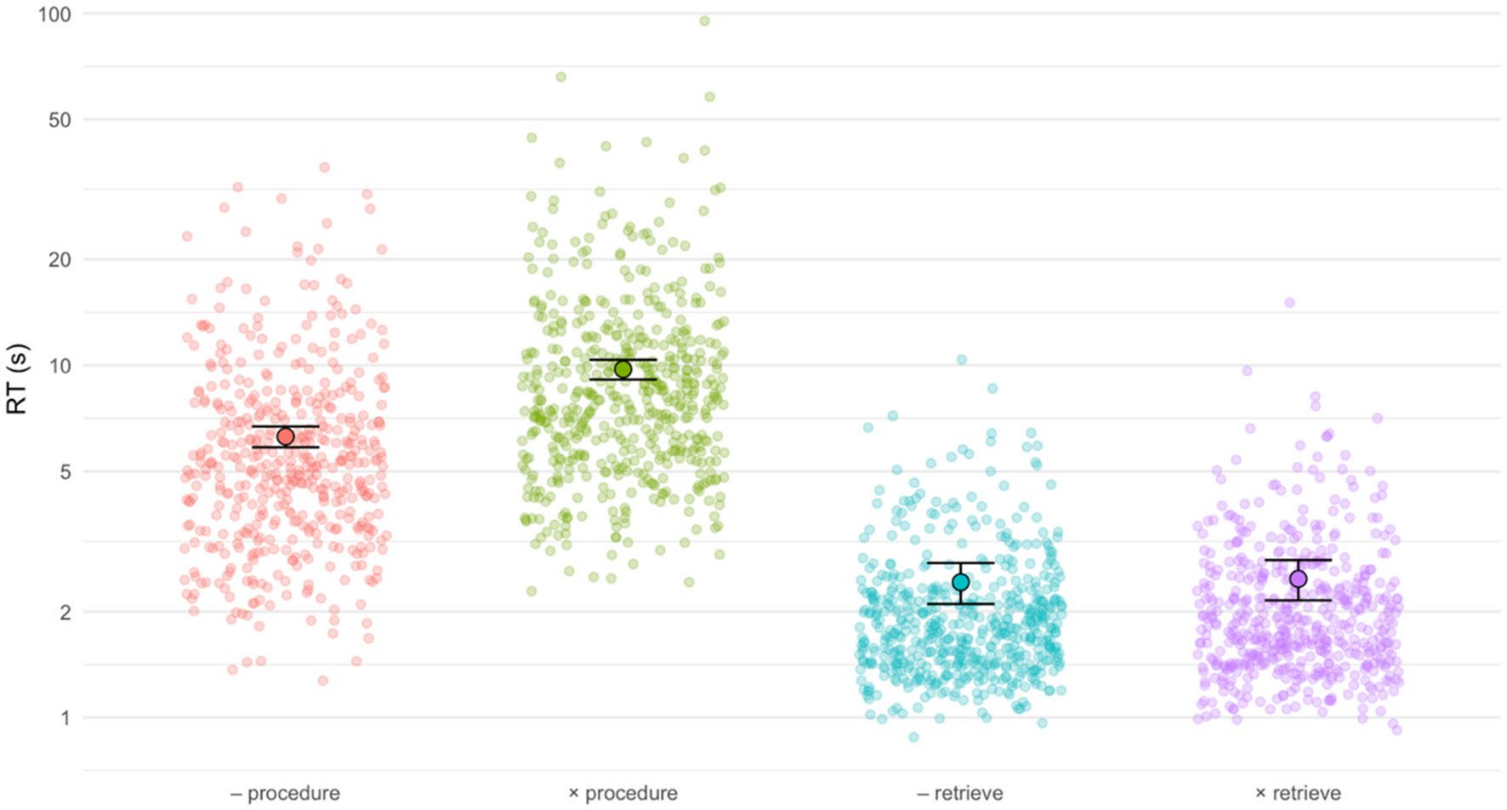
RTs for correct solutions in the four conditions. Semi-transparent dots indicate RTs for individual problems, whereas large outlined dots and associated error bars depict estimated marginal means with 95% confidence intervals. Note that the y-axis is logarithmically scaled.

The generalized linear mixed-effects model (with two fixed effects strategy and operation and one random effect participant) resulted in significant main effects of strategy (*χ*^2^(1) = 2024.54, *p* < 0.001) and operation (*χ*^2^(1) = 133.52, *p* < 0.001) as well as their significant interaction (*χ*^2^(1) = 126.27, *p* < 0.001) (see Fig. 3 for a visual representation).

Children solved subtractions significantly faster (4.07 s) than multiplications (6.17 s). Similarly, they solved retrieval problems significantly faster (2.12 s) than procedural problems (8.36 s). Tukey-corrected pairwise post-hoc tests confirm that RTs differ significantly between all pairs (all *p* < 0.001) except for retrieved subtractions versus retrieved multiplications (*p* = 0.425).

### ERD/ERS data

#### Theta band

The linear mixed-effects model for the theta (3–6 Hz) band revealed significant main effects of strategy (*F*(1, 930) = 83.17, *p* < 0.001), operation (*F*(1, 930) = 7.35, *p* < 0.01), and ROI (*F*(3, 930) = 11.29, *p* < 0.001) as well as a significant two-way interaction of strategy and operation (*F*(1, 930) = 27.47, *p* < 0.001). There was also a significant three-way interaction of strategy, ROI, and hemisphere (*F*(3, 930) = 3.11, *p* < 0.05).

**Figure 4:**
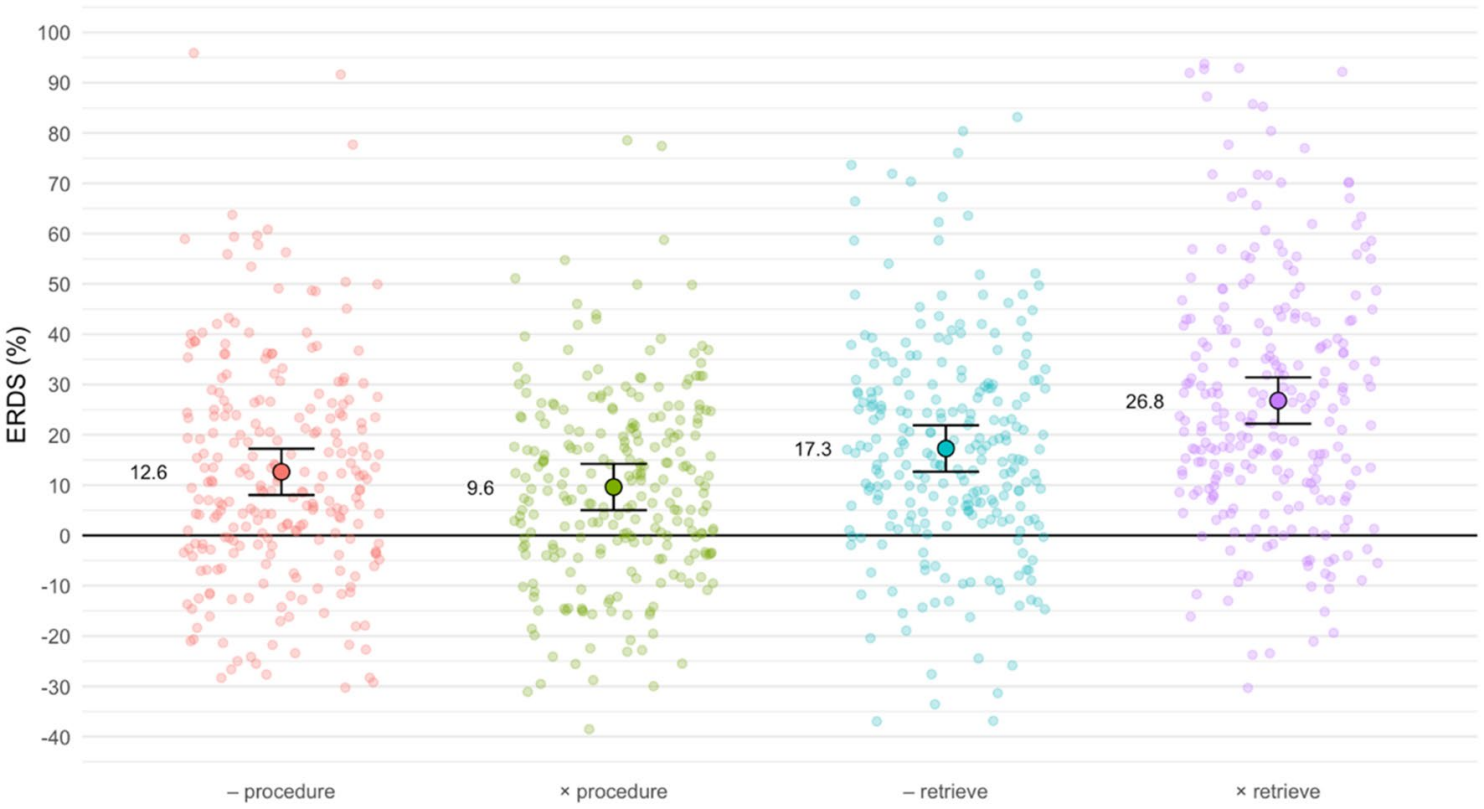
Theta ERD/ERS in the four strategy/operation combinations. Individual semi-transparent dots correspond to ERD/ERS values for individual participants and ROIs, whereas large outlined dots and associated error bars depict estimated marginal means with 95% confidence intervals. Note that we fixed the upper y-axis limit to 100% to improve visibility of the data—this removes one value exceeding this limit (117%) in retrieved multiplications.

Retrieval problems elicited significantly higher ERS (22.0%) than procedural problems (11.1%). Furthermore, multiplications were associated with significantly higher theta ERS (18.2%) than subtractions (15.0%). Analyzing the interaction of strategy and operation, Tukey-corrected pairwise post-hoc tests showed significant differences between all pairs except for procedural subtractions versus procedural multiplications (all *p* < 0.001 except for retrieved subtractions versus retrieved multiplications with *p* < 0.05). Figure 4 illustrates these differences and shows that retrieval problems were associated with higher theta ERS than procedural problems (reflected in the main effect of strategy). Within procedures, operations did not differ significantly, but retrieved multiplications elicited higher theta ERS than retrieved subtractions.

**Figure 5:**
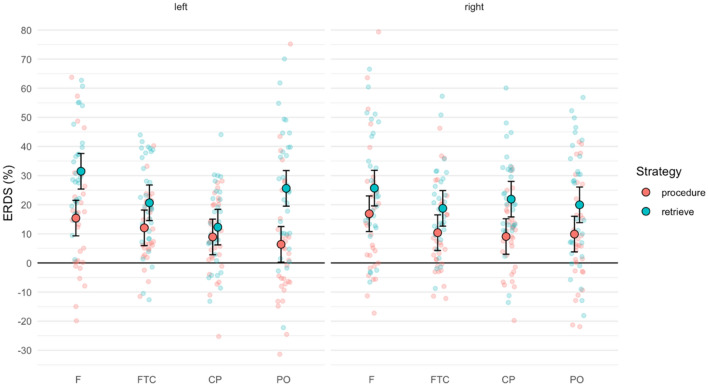
Three-way interaction of strategy, ROI (*F* frontal, *FTC* fronto-temporo-central, *CP* centro-parietal, *PO* parieto-occipital), and hemisphere in the theta band. Semi-transparent dots correspond to ERD/ERS values for individual participants, whereas large outlined dots and associated error bars depict estimated marginal means with 95% confidence intervals.

The three-way interaction of strategy, ROI, and hemisphere is shown in Fig. 5. This interaction involved topographical ERD/ERS differences depending on the three factors, resulting in 120 pairwise post-hoc tests. Focusing on differences between retrieval and procedural strategies within a given ROI, we found significant differences in left frontal (*p* < 0.001), left parieto-occipital (*p* < 0.001), and right centro-parietal (*p* < 0.05) areas (after Tukey correction).

#### Lower alpha band

In the lower alpha band (8–10 Hz), the linear-mixed effects model yielded significant main effects of strategy (*F*(1, 930) = 18.60, *p* < 0.001), ROI (*F*(3, 930) = 46.25, *p* < 0.001), and hemisphere (*F*(1, 930) = 4.41, *p* < 0.05). In addition, the interaction of operation and strategy was also significant (*F*(1, 930) = 8.41, *p* < 0.01).

Starting with the main effects, procedural problems were associated with less synchronization or more desynchronization (− 1.83%) than retrieval problems (3.48%). In other words, on average procedural problems showed ERD, whereas retrieval problems showed ERS (the mean difference is 5.31%). ERD/ERS values decreased from frontal to parieto-occipital regions (7.63%, 6.60%, − 0.39%, and − 10.54%, respectively), and they were larger in the left (2.12%) compared to the right hemisphere (− 0.47%).

**Figure 6:**
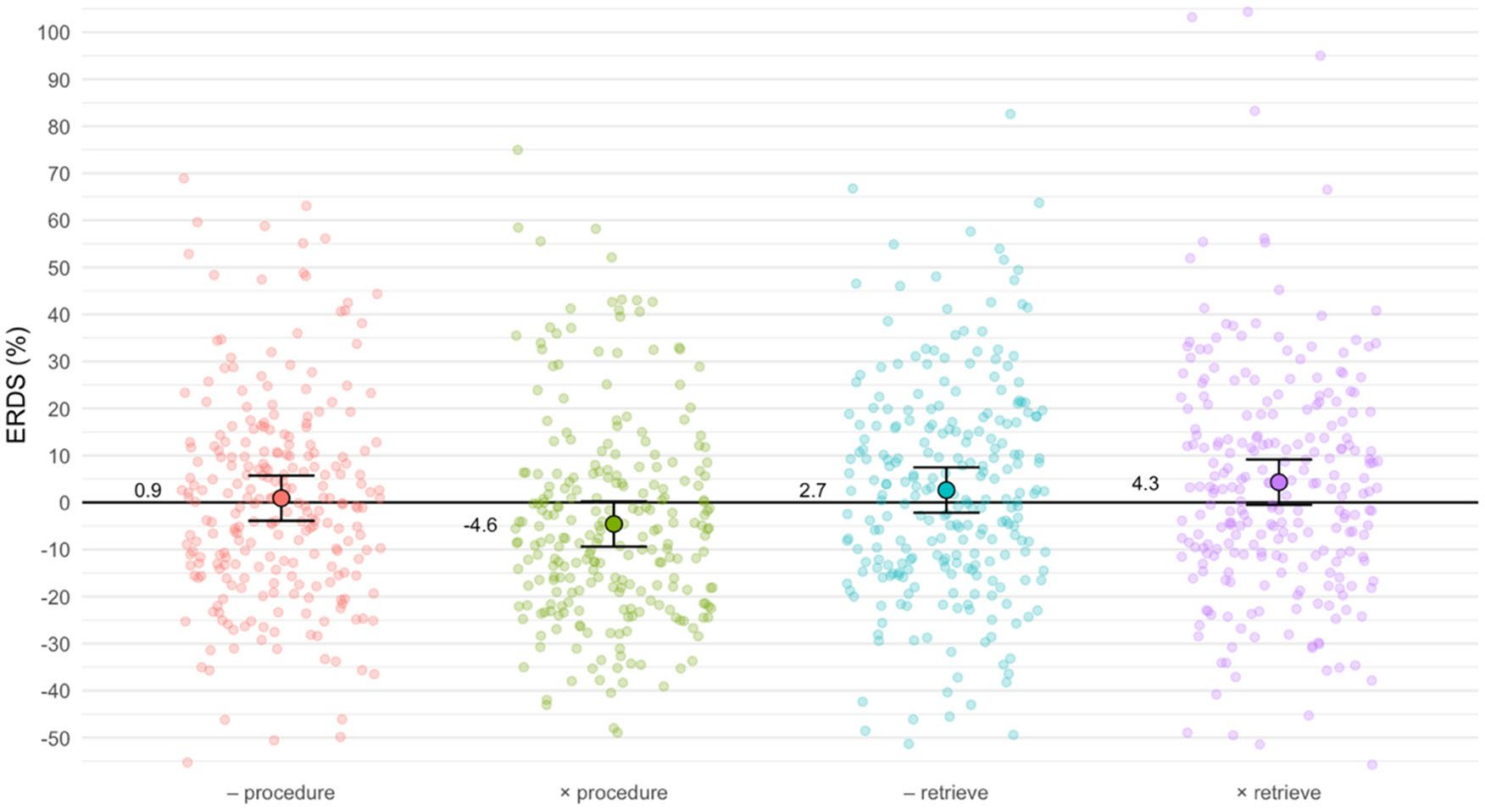
Lower alpha ERD/ERS in the four strategy/operation combinations. Individual semi-transparent dots correspond to ERD/ERS values for individual participants and ROIs, whereas large outlined dots and associated error bars depict estimated marginal means with 95% confidence intervals. Note that we fixed the y-axis limits between − 50 and 100% to improve visibility of the data. This removes three values exceeding these limits (112% in procedural subtractions, − 63.7% in procedural multiplications, and 154% in retrieved multiplications).

Figure 6 illustrates the interaction of operation and strategy. Tukey-corrected pairwise post-hoc tests showed that procedural multiplications differed significantly from all other problems (*p* < 0.01 when compared to procedural subtractions, *p* < 0.001 when compared to both retrieval problem types). There was no significant difference between operations within retrieval problems.

#### Upper alpha band

The model for the upper alpha band (10–13 Hz) revealed significant main effects of strategy (*F*(1, 930) = 7.64, *p* < 0.01) and ROI (*F*(3, 930) = 88.09, *p* < 0.001). Concretely, procedural strategies were associated with larger ERD values than retrieval problems (− 11.48% versus − 8.84%). Similar to the lower alpha band, upper alpha ERD decreased from frontal to parieto-occipital regions (− 4.20%, − 4.18%, − 9.14%, − 23.11%, respectively).

## Discussion

This study assessed fourth graders (aged 9–10 years) while solving multiplication and subtraction problems. Our primary goal was to quantify ERD/ERS patterns related to arithmetic strategy use and different operations in this age group.

We found similar behavioral patterns as those present in adults. Children solved retrieval problems faster and more accurately than procedural problems. Whereas there was virtually no RT difference between operations in retrieval problems, children took significantly longer to solve procedural multiplications than procedural subtractions—sometimes as long as one and a half minutes (see Table 5 for detailed summary statistics). After the experiment, several children reported severe difficulties with this problem type, mainly because they had not solved such large multiplications without pencil and paper before. Apparently, the stimulus material developed and validated for Flemish fourth graders could not be transferred to Austrian children in a straightforward way. Math education curricula in these two countries seem to emphasize different arithmetic competencies throughout elementary school. Therefore, our results from procedural multiplications are confounded by increased difficulty when compared to procedural subtractions. This might explain the interaction of strategy and operation on response times. We discuss this topic in more detail in the ERD/ERS paragraphs below.

Despite this limitation, we still found high agreement between problem size and subjective strategy self-reports, indicating that overall, the stimulus material elicited the expected strategies in our sample. In total, children’s strategy reports disagreed with problem size in 324 out of 2480 problems, which we excluded from our analyses. Although this decision discards a significant amount of data, it increases the validity of our problem categorization because we only consider problems where subjective and objective categorizations match.

Theta ERS is a correlate of arithmetic fact retrieval, as found in several previous studies with adults. Specifically, fact retrieval is associated with increased left-hemispheric theta ERS when compared to procedural strategies^17, 21, 46–48^. In our sample of fourth graders, retrieval problems also elicited higher theta ERS than procedural problems across both operations. We also observed a significant interaction between strategy, hemisphere, and ROI, which is compatible with patterns in adults. We found significant theta ERS differences primarily in left-hemispheric regions (frontal and parieto-occipital), but also in the right centro-parietal region (see Fig. 5).

Our results also demonstrate a clear operation effect. In general, multiplications are associated with higher theta ERS than subtractions. This main effect, however, is superseded by the interaction of strategy and operation (see Fig. 4). Retrieved multiplications are associated with higher theta ERS than retrieved subtractions, whereas there is no significant operation difference within procedural problems. Therefore, our findings provide evidence that solving multiplication and subtraction problems are associated with distinct neurophysiological patterns within retrieval problems. In other words, we found operation differences only within retrieval but not within procedural problems.

It is critical to note that neither response times nor solution accuracies differed significantly between retrieved multiplications and retrieved subtractions. As a result, the higher theta ERS finding cannot be explained by differences in problem difficulty between operations. Instead, it could reflect a genuine effect of operation within retrieval problems visible in this frequency band. Conversely, theta ERS seems to be insensitive to operation differences in procedural problems despite the significant and large RT difference in this category.

These results beg the question why retrieved subtractions are linked to lower theta ERS than retrieved multiplications. Because several studies have associated enhanced theta ERS with fact retrieval^17, 21^, one explanation for this finding is that children solved multiplications consistently via fact retrieval, whereas they used a mix of retrieval and procedural strategies for subtractions. In fact, children might find it difficult to immediately reflect on their problem-solving strategy^72^, which could explain why they reported all of these subtractions as retrieved. The resulting mix of strategies in subtractions (i.e., children solved at least some problems with a procedure) would be in line with overall decreased theta ERS when compared to exclusive fact retrieval as observed in multiplications.

Furthermore, training and learning history of these two arithmetic operations could play an important role. While multiplications are typically learned by rote in school (which means they are more likely to be solved via fact retrieval)^14^, subtractions are solved with procedures such as decrementing or calculating the solution to the associated inverse addition problem^58, 73^. In fact, Ischebeck et al.^74^ studied young adults solving multiplications (two-digit times one-digit numbers) and subtractions (two-digit minus two-digit numbers) before and after training. As expected, they found that training led to faster RTs and higher accuracies in both operations. However, whereas frontal and parietal areas showed stronger brain activation in untrained as opposed to trained problems (reflecting higher activity of general-purpose functions such as working memory and executive control), only trained multiplications elicited higher activation in the left angular gyrus. The authors interpreted this finding as a shift from procedural strategies to retrieval in multiplications, whereas solving subtractions continued to rely on procedures, but these procedures also became faster and more efficient. Thus, our finding of less pronounced theta ERS in retrieved subtractions as compared to retrieved multiplications might indicate that children predominantly retrieved the solutions to multiplication problems, whereas they solved at least some subtractions with (equally fast) procedures^73^.

Our theta ERS finding is also compatible with an fMRI study by Prado et al.^55^, who studied single-digit multiplication and subtraction in children from second to seventh grade cross-sectionally. They discovered that although multiplications and subtractions did not differ on a behavioral level, only multiplications were associated with increased neural activity in a language-related area in the left temporal cortex (which plays an important role in verbal fact retrieval). In contrast, they observed stronger activity in the right parietal cortex only for subtractions with increasing age, an area involved in the procedural manipulation of quantities^22^. Similarly, Prado et al.^52^ reported distinct neural representations of subtractions and multiplications in adults.

Alternatively, the observed theta ERS differences between retrieved multiplications and subtractions could also be explained by operation-dependent fact retrieval mechanisms. In fact, several studies provide evidence that arithmetic facts are organized differently across arithmetic operations. For example, Van Harskamp and Cipolotti^59^ report selective impairment of simple addition, multiplication, and subtraction in their study of three patients with different lesions. They argue that their findings are compatible with the assumption that arithmetic facts are stored in separate networks depending on the involved arithmetic operation, a model initially proposed by Dagenbach and McCloskey^60^ based on a case study with a single patient. The fMRI study by Rosenberg-Lee et al.^61^ supports this account by showing that in addition to pronounced individual differences, cortical activation differed across difficulty-matched problems involving the four arithmetic operations. Along the same lines, Zhou et al.^51^ found evidence that single-digit addition relies on visuospatial processing, whereas single-digit multiplication is more strongly associated with verbal processing. In summary, our theta ERS results, which showed higher values for retrieved multiplications as opposed to retrieved subtractions, can also be explained by operation-specific fact retrieval mechanisms.

Regarding theta ERS topographies, the most pronounced differences between retrieval and procedural strategies were located in the left-hemispheric parieto-occipital region (see Fig. 5). This pattern is consistent with previous EEG studies in adults, which also pinpointed the location with largest strategy differences to this region^17, 21^. The location is also in line with previous fMRI studies, which associated fact retrieval with left-hemispheric language-related areas^55^. However, our study as well as previous related studies reported results based on surface EEG, so patterns are confounded by volume conduction and therefore need to be interpreted with caution. Source identification and localization methods^75, 76^ could greatly improve spatial estimates of cortical activity in future studies, but this would likely require more than 32 channels as used in this study.

Widespread lower alpha ERD (8–10 Hz) is thought to correlate with task complexity and attention^34, 77^. In line with this hypothesis, previous studies on arithmetic reported increased lower alpha ERD for large procedural problems as compared to small retrieval problems^21, 46, 68^. Our study with children corroborates these findings. We observed stronger lower alpha ERD for procedural than for retrieval problems. We also found a decrease from frontal to parieto-occipital regions as well as slightly higher values in the left as compared to the right hemisphere. This topographic pattern is compatible with a previous study in adults^17^, which reported strongest alpha ERD at parieto-occipital sites. However, that study used a broad alpha band (8–12 Hz) instead of separate lower and upper alpha bands.

Operation differences within procedurally solved problems in the lower alpha band can be explained by increased difficulty in multiplications, which is clearly visible in the corresponding response times. In general, stronger alpha ERD is associated with increased mental effort required for more complex tasks. This is also reflected in our results: procedural multiplications were more difficult and required more effort than procedural subtractions. This explains the significantly larger alpha ERD in multiplications when compared to subtractions (see Fig. 6). However, unlike in the theta band, we cannot disentangle this difficulty effect from a potential operation effect in this frequency band.

Effects in the upper alpha band are hypothesized to be topographically more specific^34^. However, some previous studies on arithmetic problem solving found interesting effects in only one of the two bands^21^ or did not distinguish between lower and upper alpha bands at all^17^. In our study, upper alpha ERD effects were similar to those in the lower alpha band with the exception that operation differences did not emerge. Otherwise, procedural problems were associated with stronger ERD than retrieval problems, and ERD values decreased from frontal to parieto-occipital regions. In fact, we observed strongest ERD in parieto-occipital regions, which is both in line with previous studies in adults^17, 21^ as well as with the assumption that upper alpha ERD is topographically restricted to specific areas. The fact that lower and upper alpha bands sometimes do not contain distinct effects might also explain why we see a topographic pattern in both alpha bands in our study. Effects in both alpha bands overlap considerably, which further supports the notion that they do not provide distinct information in our study, but that we can rather interpret enhanced alpha ERD as a correlate for increased task demands and mental effort observed in procedural strategies.

## Conclusion

Our study explored oscillatory ERD/ERS patterns in fourth graders solving multiplication and subtraction problems. Results are generally in line with studies in adults. Children solved retrieval problems faster and more accurately than procedural problems. Whereas response times did not differ between operations within retrieval problems, we found significantly longer response times for procedural multiplications compared to procedural subtractions.

ERD/ERS patterns were generally similar to those observed in adults, namely enhanced left-hemispheric theta ERS over frontal and parieto-occipital areas for retrieval problems as well as stronger lower alpha ERD for procedural problems. Importantly, theta ERS also revealed differences between retrieved multiplications and retrieved subtractions despite the absence of behavioral differences. This could imply stronger reliance on fact retrieval for multiplications than for subtractions in this problem category, or different retrieval processes for these operations. Finally, lower alpha ERD was stronger for procedural multiplications than for procedural subtractions, which likely reflects the fact that multiplications were more difficult than subtractions.

## Data Availability

Data and analysis scripts are available at https://osf.io/5r83m/.
